# United States tea: A synopsis of ongoing tea research and solutions to United States tea production issues

**DOI:** 10.3389/fpls.2022.934651

**Published:** 2022-09-23

**Authors:** John C. D’Auria, Stephen P. Cohen, Jason Leung, Kayla Glockzin, Kyle Mark Glockzin, Jacquelyn Gervay-Hague, Dapeng Zhang, Lyndel W. Meinhardt

**Affiliations:** ^1^Metabolic Diversity Group, Department of Molecular Genetics, Leibniz Institute for Plant Genetics and Crop Plant Research (IPK), Seeland, Germany; ^2^Sustainable Perennial Crops Laboratory, U.S. Department of Agriculture-Agricultural Research Service, Beltsville, MD, United States; ^3^Department of Biochemistry and Biophysics, Texas A&M University, College Station, TX, United States; ^4^Department of Chemistry, University of California, University of California, Davis, Davis, CA, United States

**Keywords:** tea production, tea genetics, tea biochemistry, United States agriculture, tea genomics

## Abstract

Tea is a steeped beverage made from the leaves of *Camellia sinensis*. Globally, this healthy, caffeine-containing drink is one of the most widely consumed beverages. At least 50 countries produce tea and most of the production information and tea research is derived from international sources. Here, we discuss information related to tea production, genetics, and chemistry as well as production issues that affect or are likely to affect emerging tea production and research in the United States. With this review, we relay current knowledge on tea production, threats to tea production, and solutions to production problems to inform this emerging market in the United States.

## Introduction

Tea, or *Camellia sinensis* (L) Kuntze, is a sub-tropical species of evergreen shrub or small tree, in the plant family Theaceae, that is native to Southwest of China and extending to around such areas as Laos, Burma, Nepal, and Vietnam. This C_3_ plant is grown for its young leaves that are processed and used to make a water based infused beverage. This caffeine-containing non-alcoholic beverage is the most widely consumed drink in the world after water. Cultural production practices maintain this woody perennial tree in a bushy vegetative stage so that young leaves and buds can be harvested during the production period for processing into the three main tea types used by consumers: black tea (oxidized), green tea (non-oxidized) and oolong tea (semi-oxidized).

The initial use of tea as a medicinal beverage was started in China and there are reports of tea plants that are more than 1,500 years old in the Yunnan province of Southwestern China (Hara et al., 1995). Tea subsequently became popular throughout China during the Tang and Sung dynasties (618–907). The origin of tea in Japan is thought to have been derived from materials brought from China during the 9th century by Buddhist monks. Tea making changed during the Ming dynasty (1368–1,644) at which time steeping of the whole leaf became a standard practice, which was followed by a new processing method, tea leaf rolling. This rolling process was further advanced in Japan during the 18^th^ century with the Sencha method that combined steaming, drying, and rolling of the green tea leaves.

While Arab traders documented the use of tea as early as the 9th century, and Portuguese traders introduced tea to Europe in 1559; the use of tea in Europe did not become popular until 1,606 when the Dutch East Indies Company started importing tea from China and Japan. The Dutch tea drinking culture was carried to New Amsterdam in the New World. This practice continued in the colony after the English took control and renamed the colony New York in the late 1660’s. Tea popularity in England expanded when the British East India Company started importing tea in commercial quantities (Ukers, 1935; Macfarlane and Macfarlane, 2003). By the 1690’s, serving tea was a prominent practice of the New York colony. As tea grew in importance to the British empire, its demand grew, and supply limitations led to production expansions globally. Tea seeds were brought to North America by trading ships from China and by the end of the 18th century, tea could be found growing in South Carolina. Wild tea was found in Nepal and the Assam region of India in 1788 and 1823, however, those teas were not used commercially. Tea production by the British was started in the Assam region of India in 1834 with tea plants and production techniques illicitly acquired from China by Robert Fortune, a Scottish botanist, and G.J. Gordon of the East India Company. Tea production in the Darjeeling region of India started in 1841 from allegedly the same teas used in Assam (Ukers, 1935; Macfarlane and Macfarlane, 2003). The British tea production in India proved the practicality of growing tea commercially outside of East Asia and inspired attempts in the Americas to grow tea, particularly in the southern states. Based upon the preferred Chinese growing regions between the parallels of 20° and 45° N Latitude and taking into consideration the need for loam soils, Delaware, Maryland, The Carolinas, Georgia, Florida, Alabama, Mississippi, Tennessee, Kentucky, Arkansas, Louisiana and Virginia were deemed viable tea growing regions in the United States (Shepard, 1893). In 1859, 32,000 plants produced from seeds were sent to the United States Commissioner of Patents, Charles Mason, by Robert Fortune, who had been contracted to obtain the seeds from China. Unfortunately, the establishment of these tea plants was not completed and many of the plants were distributed by congressmen to their constituents throughout the Southern states.

Tea production in the United States was largely halted due to the Civil War, but started again with $5,000 and $10,000 Congressional appropriations made to USDA in 1880 and 1881, respectively, to fund a research station in Summerville, South Carolina (Klose, 1950). This research station became The Pinehurst Tea farm in 1890 when USDA funding ended, and Dr. Charles Shepard purchased the station. Over the next two decades, Dr. Shepard documented his experiments enumerating agronomic, economic, and cultural aspects of tea cultivation in various South Carolina tea gardens. Approximately 50 years later, tea cultivation studies were initiated in California (Ingbretsen, 1972). Like the Shepard studies, several test plots were established using seeds and cuttings from South Carolina sources. Following the California studies, small-scale tea growing and processing studies were conducted on the island of Hawaii. Three clonal varieties (Bohea, Yabukita and Yutaka midori) were included in the study along with the most extensive description of pest and diseases affecting *C. sinensis* in Hawaii to date (Zee et al., 2003). The California and Hawaii experiments indicated that clonal tea plants afforded uniform growth, which was deemed important for mechanical harvesting. The South Carolina and California studies concluded that labor costs associated with tea harvesting were a limiting factor toward commercial tea production. Acknowledging this reality, researchers at The University of Florida introduced the idea of growing tea in the home landscape (Crane and Balerdi, 2005).

More recently, several studies of domestically grown tea have appeared in the literature. Researchers from University Georgia evaluated alternative propagation and nursery systems (McConnaughey, 2013). In-ground, greenhouse, and container nursery systems were compared; the in-ground system was up to 10% more efficient. It was concluded that approximately 400,000 liners would have to be sold annually in order to sustain centralized production, which would translate to approximately 100 acres of tea being planted each year. It is estimated that fewer than 100 acres of tea are currently planted in the US and many of the growers are relatively small operations of less than 10 acres. Equally important considerations of scale include the number of mature plants in North America available for propagation and knowledge of the plant characteristics to assure high quality tea production. To address these issues, Bi and co-workers screened nine tea cultivars grown at Mississippi State to assess both leaf quality and plant growth (Zhang Q. et al., 2020). All were found to possess attributes required of green tea production. Internationally, tea is grown in at least 50 countries and the top ten tea producing countries (includes both green and black tea) according to FAOSTAT 2020 are China 2.98 M tons (1), India 1.42 M tons (2), Kenya 569,500 tons (3), Sri Lanka 278,489 tons (4), Turkey 255,183 tons (5), Vietnam 240,493 tons (6), Indonesia 138,323 tons (7) Myanmar 126,486 tons (8), Iran 84,683 tons (9), and Japan 69,800 tons (10).

In this literature review, we present a comprehensive review of tea-related issues, including background information, the genetics, genomics, and chemistry of tea, and tea production issues from global tea research. Understanding this research is critical for understanding issues that are likely to be important in the emerging tea industry in the United States.

## Tea production, genetics and chemistry

### Production

There are several stages of tea production beginning with propagation, cultivation and all agricultural activities required for plant growth, followed by harvesting of the leaf, and finally processing of the leaf. *C. sinensis* is an evergreen shrub or tree, that can reach up to 17 meters if not pruned (Martin et al., 1997). Tea plants will take at least 3 years before they are mature enough for harvesting. Typically, during this time the trees are pruned to the desired shape and height to maintain the tea plants in a harvestable shrub form (Martin et al., 1997). Tea plants are normally grown in well-drained acidic soils that have a high organic matter content. The plants need to be protected from strong winds that can damage the tender young leaves. Terroir, or the environment where tea is grown, affects the flavor, thus elevation, soil type, shading, temperature, and rainfall can impart unique characteristics to the final tea. The genetic diversity, the type of tea being processed and how it is harvested will also affect the final flavor.

**Figure 1:**
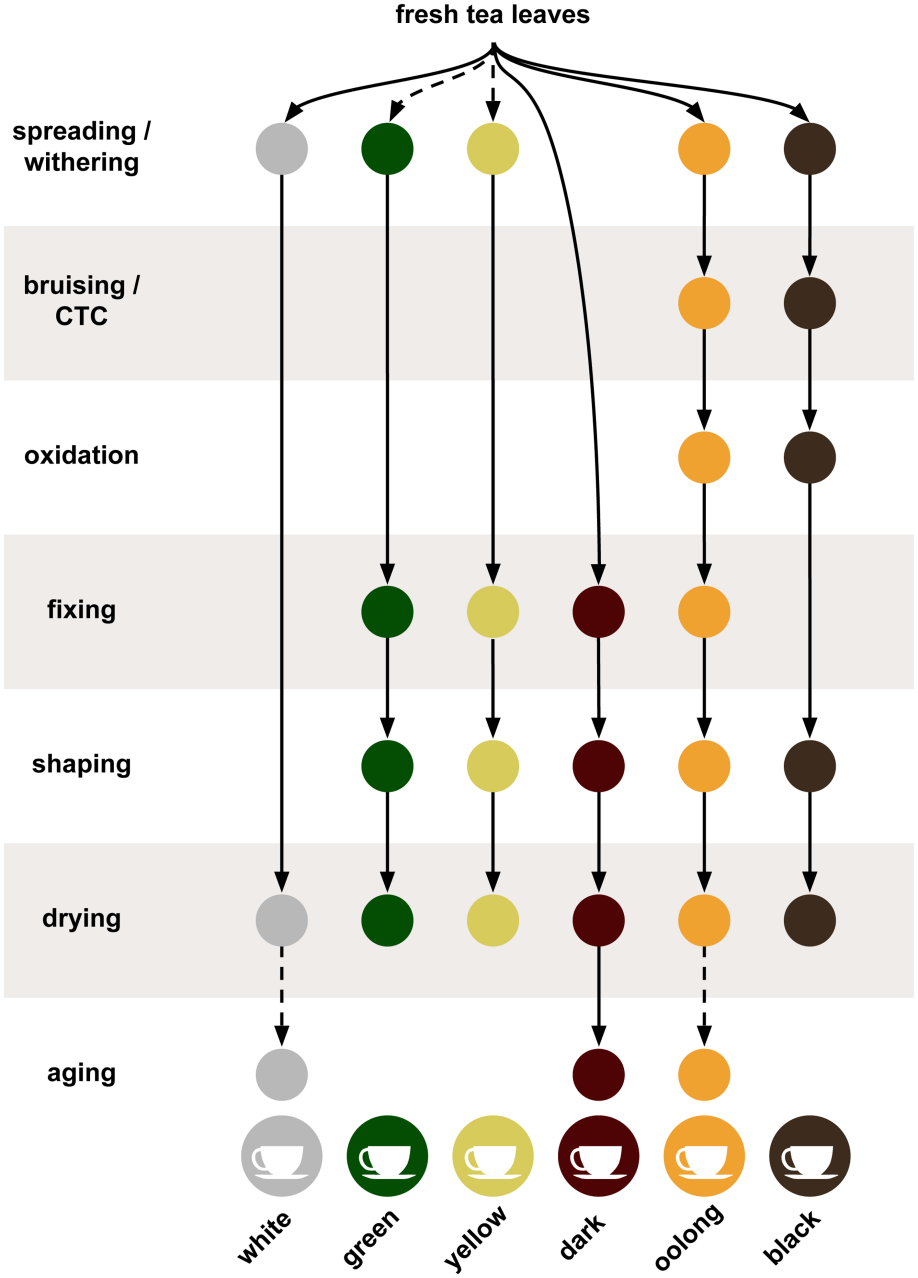
The tea production process summarized for the six different styles of tea. Solid lines indicate steps always used while dotted lines indicate steps sometimes or optionally used.

Tea leaves are harvested by plucking new leaves and terminal buds from the tips on the branches. Alternatively, mechanical trimmers can be used to cut the new flushes from the plants. These harvesting techniques aim to remove the buds and several young immature leaves, for subsequent processing while stimulating the growth of dormant terminal buds thus forming new shoots and leaves. Successive harvests are typically done during the growing period and are conducted at intervals that range from 4 to 14 day, depending on the growth rate of the plants. After removing the tea leaves, they are normally sorted before processing. Post-harvest processing is summarized in Figure 1 for multiple tea types. At harvest, the tea leaves can have a moisture content of 75–83%, which is reduced during the next step in the process called wilting or withering (Tomlins and Mashingaidze, 1997). In the withering step, the leaves are spread out onto racks under controlled drying conditions; the leaves will soften and the moisture content of the leaves drops to less than 70%. However, the actual moisture percentage will vary depending on the crafting style (Das et al., 2017). Without withering, the later steps can result in cooked leaves instead of the desired dried leaves. White teas (minimally fermented and processed), oolong teas (semi-fermented), and black teas (fermented) all undergo withering, whereas green and yellow teas (minimally fermented) either undergo short periods of withering or none at all. Dark teas (post-fermented) are usually not withered (Ye et al., 2022).

Oolong and black teas undergo bruising processes during or after withering that roll, crush, and twist the leaves to break the cell walls open, releasing the oxidizing enzymes, exposing chemical components to oxygen, and releasing volatiles (Hu et al., 2018; Wong et al., 2022). This bruising process must be uniformly done for the entire batch of leaves, therefore some have replaced this method with chopping to acquire the desired bruising. The latter technique is sometimes called crush, tear, and curl (CTC) and results in a shredded granular leaf particles (Pou et al., 2019). The bruised leaves are then subjected to another drying step, which continues the oxidation and enzymatic activity of the cellular contents turning the leaves brown. These enzymatic activities are primarily associated with polyphenol oxidases and peroxidases (Subramanian et al., 1999). Oolong teas are produced by limiting the oxidation step while black teas are fully oxidized. Green, yellow, white, and dark teas skip the oxidation step (Xu et al., 2018). After the bruising and oxidation step, teas are fixed by heating, which stops the oxidation and enzymatic reactions thus halting the browning and preserving any green color. The fixing step, also known as de-enzyming or kill-green, is used in all teas except black and white teas, which are fully oxidized or combine the fixation and drying steps, respectively (Chen et al., 2019). The fixation step can be done with steam, as in Japanese green teas, frying in a wok, as with Chinese green teas, or in a rotating drum. Depending on the crafting style of the final product, each method will impart a different taste to the tea (Ukers, 1935; Langat et al., 2015). Some teas use an additional step called rolling or shaping, which releases enzymes and breaks down components of the leaves (Naheed et al., 2007).

Drying is the final step for most teas and removes residual moisture from the leaves and stabilizes the tea for storage. Depending on the method or temperatures used, drying can also impart flavor to the tea product (Teshome, 2019). Lastly, the flavor of all dark teas, such as Pu-erh, benefit from aging over periods of months or years, while certain white and oolong teas can see improvements as well (Qi et al., 2018; Cheng et al., 2021; Hong et al., 2021; Zhang Q. et al., 2021). Dark teas continue to undergo fermentation, but distinguish themselves in that the post-processing fermentation occurs both endogenously and exogenously *via* microbes such as Aspergillus luchuensis (Hong et al., 2013).

### Altitude

The elevation at which tea is grown has a marked influence on the quality, chemical composition and delicate changes to the taste of tea (Owuor et al., 1990; Kfoury et al., 2018). These alterations in the flavor attributes are associated with the microclimate changes of elevated tea, which result in more precipitation, longer periods of mist and or dew, alterations in the amount and quality of sunlight and greater fluctuations between the day and nighttime temperatures. Other locations that mimic these temperature fluctuations have also been associated with high quality flavorful teas. In Hawaii, half-acre planting was established at three different elevations, Waiakea (600 ft), Mealani (2,800 ft) and Volcano (4,000 ft). Tea plants grown at Waiakea grew slower than at the higher elevations. Time to harvest for all three locations was 18–20 months, and processed green and oolong tea from the higher elevations was superior in quality (Zee et al., 2003).

### Genetic diversity

*Camellia sinensis* is a diploid (2n = 30) with a genome size originally estimated to be 4.0 Gbp (Tanaka et al., 2006). The genome of tea has been sequenced revealing a more accurate genome size of 3.1 Gbp (Xia et al., 2017; Wei et al., 2018). Tea has been placed into the plant family Theaceae and into two subspecies, var. *sinensis* and var. *assamica*. Before the establishment of genotyping, the primary criteria used to classify individuals into subspecies were purely morphological and included leaf size, flowers, and branching characteristics (Mukhopadhyay and Mondal, 2017). The *assamica* types have large (15 to 20 cm long), thin glossy leaves, borne on small trees with robust branches that are sensitive to environmental stresses such as drought and cold, whereas *sinensis* types have small leaves (3 to 6 cm long) that are erect and purple when young and grow into large shrubs with thick, hard, leathery leaves that can withstand environmental stresses (Mukhopadhyay and Mondal, 2017). The separation of the subspecies is supported by *matK* chloroplast nucleotide sequence polymorphisms, as well as simple sequence repeats (SSRs) identified *via* the more recent comparison of whole chloroplast genomes (Katoh et al., 2003; Meegahakumbura et al., 2018; Rawal et al., 2021).

The center of origin for *C. sinensis* is in Southwestern China, with modern cultivation spread across latitudes spanning from 45° North to 34° South (Xia et al., 2020). The speciation of tea appears to be the result of the rise of the Tibetan plateau which would have separated the founding population, providing isolation where differentiation could occur. The Yunnan-Guizhou plateau has been proposed as the area of divergence for the *assamica* and *sinensis* subspecies (Yao et al., 2012). This can be visualized by separation of the large leaf Indian Assam tea, found in eastern India from the Small leaf Chinese tea, found in western China. These subspecies have been calculated to have separated from each other somewhere between 0.38 to 1.54 million years ago (Wei et al., 2018). The current view of the genetic diversity of tea supports three main populations of tea: Indian Assam, Chinese Assam and Chinese tea (Wambulwa et al., 2016). This view is strongly supported by the recent resequencing of 81 tea accessions from a variety of different geographical origins (Xia et al., 2020). Intriguingly, this study was also able to show that there was an increase in genetic diversity of cultivated tea accessions when compared to wild varieties. Robust single nucleotide polymorphic assays have greatly improved our ability to access the genetic diversity of teas and allow the analysis to be conducted on a single tea leaf, regardless of the type of processing (i.e., green, oolong or black; Fang et al., 2014, 2016). This methodology in combination with the aforementioned chloroplast genome comparisons not only allows the assessment of genetic diversity but can be used to authenticate varieties for quality control.

### Tea chemistry

**Figure 2:**
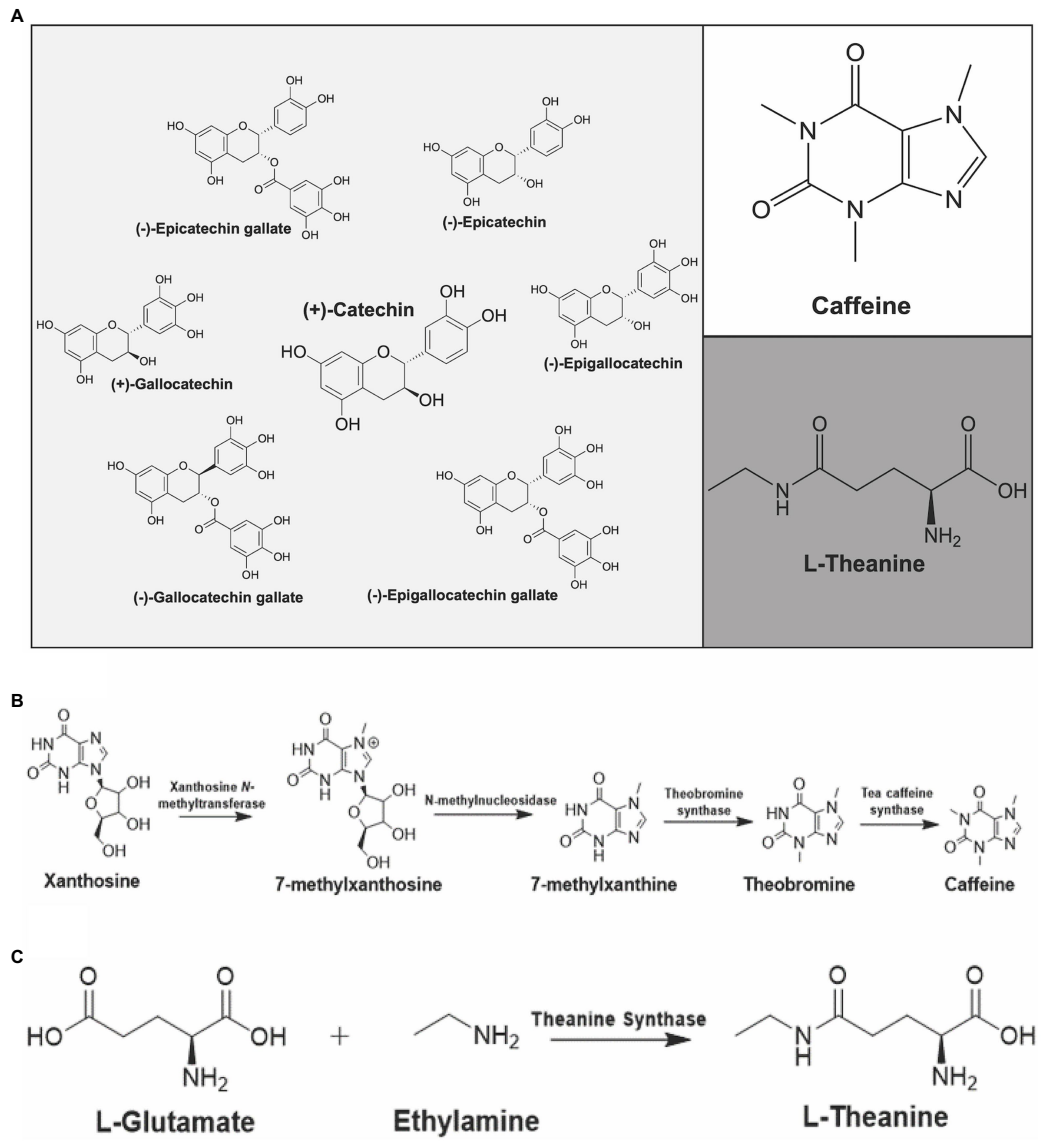
Major constituents of *Camellia sinensis* and their biosynthetic pathways. **(A)** Catechins (left), caffeine (upper right) and L-theanine (bottom right) are shown. Biosynthetic pathways of **(B)** caffeine and **(C)** L-theanine in *C. sinensis*.

The wide-ranging health benefits of tea are associated with its bioactive, secondary metabolites (da Silva Pinto, 2013; Fang et al., 2021). The major constituents associated with tea are caffeine, catechins, theanine and other free amino acids (Figure 2A; Tai et al., 2015; Zhang S. et al., 2018). Green leaf volatiles are also notable metabolites in the aroma of tea and these compounds are classified as secondary metabolites (Tai et al., 2015; Ono et al., 2016; Zhang S. et al., 2018). The sedentary nature of plants requires them to produce specialized compounds, or secondary metabolites, that fight off biotic and abiotic environmental factors (Kim et al., 2016). Specifically, these secondary metabolites play a key role in the flavor, aroma, and overall health benefits of tea (Li et al., 2015).

Caffeine is classified as a purine alkaloid and behaves as a plant defense compound due to its anti-herbivory properties (Zhao et al., 2020). Purine alkaloids are nitrogenous compounds containing a fused five-membered and a six-membered ring. Purine alkaloids are known for having certain pharmacological effects (Koshiishi et al., 2001). Specifically, caffeine is a neurostimulant that has been implicated in influencing mood, sleep, and cognitive behavior (da Silva Pinto, 2013). As little as a single cup of tea contains enough caffeine to have indicative alertness and psychomotor effects (Rogers et al., 2008). Black tea, on average, contains more caffeine in comparison to green tea, due to different cultivars used for black tea versus green tea (Astill et al., 2001). There is approximately 47.5 mg of caffeine per gram of tea bud and 30.5 mg of caffeine per gram of tea leaves (Tai et al., 2015). In both tea and coffee, the biosynthetic pathway of caffeine contains several *N-*methyl transferases (Figure 2B; Li et al., 2015). All of the *N-*methyl transferases involved in the pathway for both coffee and tea are part of the SABATH family of enzymes and utilize SAM (S-adenosyl-methionine) as a methyl donor (Ashihara et al., 2008; Li et al., 2015). The genome of *C. sinensis* var. *sinensis* contains a total of 32 SABATH genes, which are classified into three groups based on motif structure (Guo et al., 2020). Contrary to coffee, the first two *N*-methyl transferases in tea accumulate in young leaves and shoots, while the enzyme TCS (tea caffeine synthase) is present in both young leaves and mature leaves. This is indicative that caffeine biosynthesis in tea plants begins in young leaves but can be completed in either young or mature leaves (Ashihara et al., 2008; Li et al., 2015). Caffeine levels of nine tested Mississippi cultivars over three seasons consistently showed highest levels in summer (3.49–2.67%), followed by spring (2.92–2.06%) and then fall (1.53–2.46-1.53%). Varietal differences were also observed, however the seasonal trend was similar across varieties (Zhang Q. et al., 2020).

Theanine and caffeine are the major components affiliated with the taste of tea. Caffeine is associated with the bitter taste of tea and theanine is responsible for the unique taste known as “umami” (Zhang S. et al., 2018). L-theanine is a unique, non-proteinic, free amino acid derived from the amino acid glutamate (Figure 2C; Ashihara et al., 2008); theanine is a recognized antagonist against caffeine that is an effective compound for the treatment of individuals experiencing caffeine-induced paralysis. In addition, theanine is also effective as an inhibitor of caffeine-induced elevation of blood pressure (Rogers et al., 2008). The total content of theanine in tea leaves and buds is estimated to be around 1–3% (Tai et al., 2019; Huang et al., 2022). This concentration of theanine in the tea plant can still produce substantial physiological effects because it is quickly absorbed in the bloodstream following ingestion (Koshiishi et al., 2001). A wide array of positive medicinal properties has been ascribed to theanine including its promotion of relaxation and improving concentration. In addition, theanine has been implicated as an anti-tumor agent, and has been studied for the prevention of cardiovascular and cerebrovascular diseases (Liang et al., 2015). Lastly, theanine is also implicated in improving the immune system and suppressing body weight increases as well as the accumulation of fat. Besides tea plants, the only other known source of L-theanine is *Xerocomus badius*, the bay bolete mushroom (Mu et al., 2015).

The biosynthesis of L-theanine in tea begins with the amino acid L-glutamate (Figure 2C; Chen et al., 2021). It is known that mature plants are the highest producers of L-theanine, with the biosynthetic pathway being the most active in the roots. Once made, L-theanine is translocated to developing leaves *via* the phloem. Recent reports have described the biosynthesis of theanine from a tea-associated endophyte suggesting that microbial partners may also assist in the biosynthesis of theanine (Xie et al., 2020). Studies focused on measuring total amino acid content among different varieties and seasons found that theanine content was highest in the fall for all varieties tested (Zhang Q. et al., 2020). In addition to theanine, arginine and glutamine concentrations are also positively correlated with price and tea quality (Kato and Suzuki, 1971; Mukai et al., 1992; Goto et al., 1994; Wang and Ruan, 2009; Miyauchi et al., 2014).

**Figure 3:**
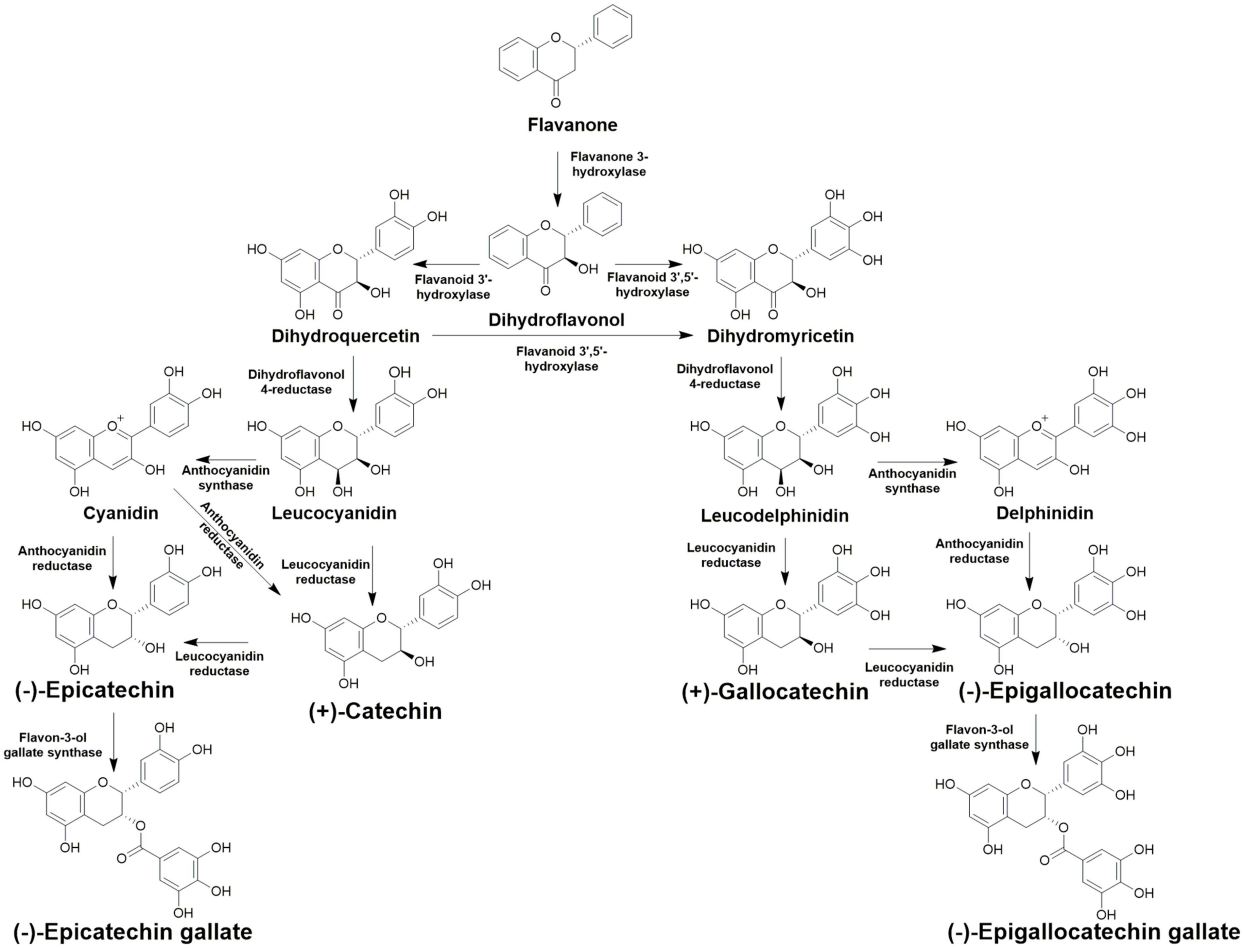
Catechin biosynthetic pathways in *Camellia sinensis*.

Flavonoids have a wide array of functions for the plant, and health benefits for consumers. The core structure of flavonoids is derived from the amino acid phenylalanine (Li et al., 2015). Catechins are classified as flavan 3-ols, a subcategory of flavonoids and can also be categorized as polyphenolic compounds. Catechins are known for their ability to protect plants from ultraviolet light and phytopathogens (Li et al., 2015). For consumers, catechins are acknowledged for their antioxidant properties, and are thought to be one of the major cardioprotective and anti-tumor components of *C. sinensis* (Musial et al., 2020). There is an abundance of catechin and catechin derived compounds present in tea, which are usually described as total catechins (Tai et al., 2015). Total catechins are defined as catechin, gallocatechin, gallocatechin gallate, epicatechin, epigallocatechin, epigallocatechin gallate, and epicatechin gallate (Figure 2A). The biosynthesis of each catechin is complex, however known flavonoid biosynthesis pathway genes have been identified and studied in tea (Zhang Y. et al., 2018). Many of the enzymes present in the biosynthetic pathway are promiscuous and can utilize multiple different substrates and yield multiple different catechins (Figure 3; Zhang et al., 2016). Catechins remain intact during green tea production. However, the bruising steps used in black tea preparation promote enzymatic oxidation of catechins, thus lowering their overall content in black tea (Astill et al., 2001). Catechins also contribute to the color and taste of tea plants (Zhang et al., 2016). The total polyphenol content of several varieties studied was highest in summer for eight of the nine cultivars tested, while the remaining cultivar was highest in spring (Zhang Q. et al., 2020). In that same study, total polyphenol content was lowest in fall across all varieties tested.

**Figure 4:**
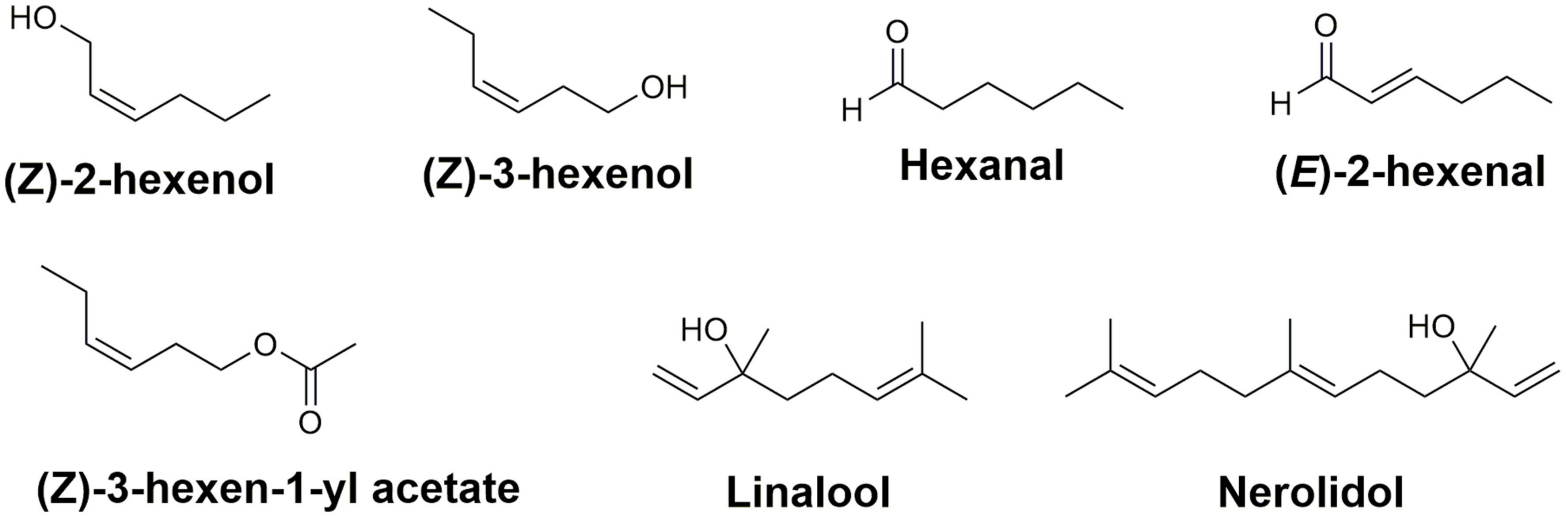
Green leaf volatiles and terpenes contributing to the aroma and flavor profile in *C. sinensis.* Collectively, these compounds are responsible for the green, grassy, sweet, and fruity aroma of different types of teas.

While green leaf volatiles are not considered to be a major component of tea, they are fundamental in developing the aroma of tea (Ono et al., 2016). Over 600 volatile compounds have been associated with tea aroma (Zheng et al., 2016b); the content of volatile compounds is dependent on the materials and methods used during the tea processing steps. Green leaf volatiles (GLVs) are 6-carbon molecules emitted when a plant is wounded mechanically (D’Auria et al., 2007). In tea, these 6-carbon volatile alcohols are the alcohols (*Z*)-2-hexen-1-ol and (*Z*)-3-hexen-1-ol, the aldehydes hexanal and (*E*)-2-hexenal, as well as the ester (Z)-3-hexen-1-yl acetate (Figure 4; Ul Hassan et al., 2015).

In addition to the green leaf volatiles, terpenes also play an important role in tea aroma and stress tolerance. Specifically, the terpene alcohols linalool and nerolidol (Figure 4) are major constituents in the volatile headspace of black and oolong teas and their storage seems to involve storage *via* the non-volatile glycosides (Mizutani et al., 2002; Liu et al., 2018). The terpene synthase and UDP-glucosyltransferase families in tea have been amplified in number *via* gene duplication and with several gene clusters co-locating with known caffeine biosynthesis related genes (Xia et al., 2020). Collectively, these volatiles are the leading contributors of the sensory components responsible for the green, grassy, sweet, and fruity aroma of different types of teas. Each of these secondary metabolites are necessary for developing the enticing aroma and rich flavor of all varieties of tea. More importantly, the major constituents (caffeine, catechins, and theanine) are responsible for the comprehensive health benefits associated with tea (Higdon and Frei, 2003; Cooper, 2012; Türközü and Şanlier, 2017). The content of tea volatiles in processed US teas has yet to be reported.

### United States production zones

In the United States, tea can be grown in the USDA Hardiness zones 7, 8, 9, and 10 where the temperature ranges from 21 to 29°C (70 to 84°F) and where there is sufficient rainfall of 150–250 cm/year (59–98 inches/year; USDA Agricultural Research Service, 2012; Duncan et al., 2016). Tea plants will decrease shoot growth and new flushes when temperatures fall below 13°C (55°F), while high soil temperature during the day and low soil temperature at night will induce flowering and reduce vegetative growth (De Costa et al., 2007). There are several cold tolerant tea clones that are claimed to survive at Hardiness zone 6b (Extension Gardener: *Camellia sinensis*, 2021).

The recent surge in artisan teas has increased tea production in the United States, with tea production occurring on more than 60 farms across 17 states (American Specialty Tea Alliance,^1^ The US League of Tea Growers).^2^ Producing tea under suboptimal growth conditions may cause further stresses to the plants. Tea plants that are exposed to abiotic and biotic stresses produce a series of additional phytochemicals. The concentrations of these metabolites vary depending on the genetic, environmental, and culture conditions (Li et al., 2007; Adnan et al., 2013; Ahmed et al., 2013). Therefore, production issues have a direct impact on the quality of the final tea product.

### United States teas and origins

The origin of tea produced in the United States have multiple backgrounds with most coming from either China or India. Since most of the teas commercially grown in the United States are derived from the South Carolina USDA Pinehurst Experimental Tea Station, historical records were evaluated to attempt to identify their ancestries. Dragon pool or Loong Tsin tea (current name Longjing = Dragon well), which was grown in the South Frazer tea gardens, was acquired in 1892 from seed harvested from celebrated gardens near Hangchow, the capital of Chekiang province in China, now Hangzhou Zhejiang (Shepard, 1893). Tea from the Rose Gardens are reported to be Assam-hybrid teas. Tea germplasm maintained by USDA and University of Hawaii are primarily comprised of Chinese and Japanese varieties. Bohea (Wuyi tea from Fujian China) is a variety suitable for both oolong and black tea production while the Japanese varieties Yabukita and Yutaka midori are suitable for both green and oolong teas (Zee et al., 2003).

### Tea in the genomics era

Advances in genomics and transcriptomics have enhanced our understanding of the genetic basis of stress responses, metabolism, and aroma and flavor in tea, while allowing for new developments in molecular breeding. The first draft assembly of *C. sinensis* var. *assamica* (Xia et al., 2017) revealed that the relatively large size of the tea genome was explained by the repetitive nature of the sequence, a large majority of which consisted of transposable elements. This genome also demonstrated that caffeine biosynthesis evolved independently in tea, with a distinct pathway from cacao and coffee caffeine biosynthesis. Successively better genomes of both *C. sinensis* varieties *assamica* and *sinensis* have been released and each subsequent genome release has led to the discovery of important genomic features, e.g., identifying the genomic signatures of artificial selection (Xia et al., 2020; Zhang X. et al., 2021), or the impact of structural variation and gene family expansion on the unique aroma of an important oolong cultivar (Wang P. et al., 2021). Modern tea genomes now reach chromosome level contiguity and are approximately 3.1 Gb in size, slightly lower than the estimates of 3.5–4.0 Gb suggested by previous cytogenetic work. To date, 17 total tea genomes have been published and made available on databases such as NCBI and NGDC, including 7 *C. sinensis* var. *sinensis* assemblies, 5 *C. sinensis* var. *assamica* assemblies, and various hybrids and wild teas (Wei et al., 2018; Xia et al., 2019, 2020; Chen et al., 2020; Zhang Q. J. et al., 2020; Zhang W. et al., 2020; Wang P. et al., 2021; Zhang X. et al., 2021). With these groundbreaking new resources, researchers are beginning to understand what governs the complex traits important to both growers and consumers, where previously, this was challenged by tea’s outcrossing tendency and long lifecycle. For example, genomic prediction and genome-wide association studies (GWAS) have been employed with moderate success to correlate single nucleotide polymorphisms with the catechin EGCG and total caffeine content (Yamashita et al., 2020). GWAS has also been used to discover sites controlling spring bud flush timing, an important factor in pest avoidance and flavor in prized early spring teas (Wang et al., 2019).

Researchers have also taken advantage of technologies that sequence mRNA and capture the content and quantity of those transcripts, collectively called the transcriptome. The first comprehensive transcriptome of tea identified 127,094 unique transcripts, providing an important tool for researchers interested in studying differentially expressed transcripts under a vast array of experimental conditions (Shi et al., 2011). Advances in modern sequencing technologies have allowed researchers to generate increasingly higher quality transcriptomes that can resolve alternative splicing events (Xu et al., 2017; Qiao et al., 2019; Wang F. et al., 2021). There has been great interest in transcriptome studies of tea and its wild and cultivated relatives over the past decade, with over 660 Datasets available on the NCBI Gene Expression Omnibus to date. Recently, transcriptome studies have been used to identify candidate genes involved in cold acclimation and stress (Wang et al., 2013; Li et al., 2019; Samarina et al., 2020), drought tolerance (Liu et al., 2016; Wang W. et al., 2016; Samarina et al., 2020), heat shock (Seth et al., 2021), aluminum stress (Huang et al., 2021a), salt stress (Zhang et al., 2017; Wan et al., 2018) and more. Researchers have also used transcriptomics to better understand how genes involved in secondary metabolite pathways are differentially expressed across growing practices and time, e.g., by observing the effect of red-light withering on amino acid and theaflavin biosynthesis in black teas (Li et al., 2021), or by tracking anthocyanin degradation across the seasons (Maritim et al., 2021). The cumulative effect of all these efforts with transcriptomics is the construction of a large reservoir of target genes and gene networks to potentially inform tea improvement.

## Tea production issues

### Water availability

Drought stress is a major tea production problem and can affect most growing regions at least some time throughout the year. Globally, drought is responsible for yield reduction of 14 to 20% and the death of 6 to 19% of the tea plants (Hajiboland, 2017). Young tea plants are particularly susceptible due to drought due to under-developed root systems (Karunaratne et al., 1999). Drought stressed plants typically have reduced chlorophyll concentrations, which subsequently reduces photosynthesis and increases levels of malondialdehyde which indicates oxidative stress, further reducing growth (Guo et al., 2017). Drought stress reduces the total polyphenols, free amino acids, total flavanols, catechins, caffeine, theanine, and starch, while ABA, ethylene, salicylic acid, mannitol, trehalose, and sucrose increase in the leaves (Liu et al., 2016; Wang W. et al., 2016). Transcriptome analysis has revealed that there is a plant specific gene response to both drought and cold, suggesting that tea has a combined response to these stresses (Zheng et al., 2016a). Tea harvested during the spring drought was 50% lower in production but had 50% higher levels of catechin and methylxanthine (Ahmed et al., 2014). Drought tolerant clones and drought tolerant root stocks are two approaches that are being used to combat persistent drought conditions. One method identified for the selection of drought tolerant tea clones is to screen for higher polyphenol and catechin contents during drought stress, which appears to be a common mechanism in tolerant lines (Cheruiyot et al., 2007). Irrigation as a backup for inadequate rainfall was first suggested for US tea production by Dr. Charles Shepard in 1893 (Shepard, 1893). The use of irrigation can be used in most environments provided there is sufficient high quality ground water, and the use is economically feasible.

Instead of not enough water, some potential tea-growing regions of the United States are susceptible to flooding and too much rain. During flooding, water saturates the soils and air is no longer available to the root (Dat et al., 2004). Under these conditions, roots become dependent on the gas exchange with the aerial portion of the plant and may suffer hypoxia, limiting the O_2_ required for respiration available to the roots. In water-logged soil conditions, the soil pH increases to a neutral level (7.0) which interferes with the mineral uptake of the plant, particularly nitrogen and phosphorus (Patrick and Mahapatra, 1968). The selection of flood-resistant tea clones has helped some global tea production regions (Duarah et al., 2012). Symptoms associated with flooding in tea are stunted plants, defoliation and death of the plants.

### Shading

Shading is the act of limiting sunlight to the tea plant (75–95%), typically by covering the plants, which alters the photosynthesis of the plant, changes temperature, transpiration rates, and the morphology and chemical composition of the harvested leaves (Sano et al., 2018). Leaf thickness increases and leaf weights are reduced under shaded conditions. In tea, shading is used to manipulate the growth of the tea leaves and harvested leaves and is typically only done for high grade green teas. The length of shading for the tea plants varies from 9 to 20 days prior to harvest, and the morphology of the harvested tea shows a tendency for longer shoot lengths, more leaves per shoot, and larger dark green leaves. Non-gallate catechin levels are reduced by shading while caffeine, theanine, asparagine, glutamine, glutamic acid, tryptophan, phenylalanine and aspartic acid are increased (Wang et al., 2012; Lee et al., 2013; Sano et al., 2018; Yang et al., 2021).

### Frost

Tea production in the United States primarily occurs in the temperate zones, which can subject this semi-tropical plant to frost damage. During dormant non-growth periods, tea plants can survive several hours of −10°C (14°F) without permanent damage. However, once new flushes have started to grow, the cold tolerance of the plant is greatly reduced and temperatures as low as −2°C (29°F) can cause significant frost damage. Generally, there are two types of frost damage that occurs on the leaves: a white frost damage that occurs on the leaf surface and turns the leaves white, and black frost, which freezes the leaves without any change in the color of the leaf. In some regions of the world frost damaged leaves are harvested to produce a black or green tea that is referred to as frost tea. The effects of low temperatures upon tea grown in South Carolina were noted by Charles Shepard (1899). Plants that were fully covered in snow before the cold front fell upon them survived well, presumably because they were brought into hibernation by the earlier snow fall (Shepard, 1899). Plants with exposed leaves were severely damaged by the cold front and had to be pruned in the spring. However, this episode did not impact the long-term production; in many tea-growing regions of the world, tea plants are periodically rigorously pruned to rejuvenate the plant and increase lifetime yield (Martin et al., 1997).

### Soil pH

Soil pH under most tea growing is typically low (pH = 4.5–6), resulting in acid soils. Fertilization and high rain fall can further reduce soil pH. In tea, liming of soil in not normally recommended unless the soil pH falls below 4.0 because the high calcium levels from liming can reduce potassium uptake. In California, sulfur was used to decrease the soil pH.

### Fertilizer and nutrient deficiencies

Harvested tea leaf yields under favorable climate and nutrient conditions can reach 4 to 5 tons/ha/year and under exceptional conditions and have reached 6.5 tons/ha/year (Hajiboland, 2017). The most important nutrients found in tea flushes are nitrogen (N), potassium (K), calcium (Ca), phosphorus (P), sulfur (S), magnesium (Mg), and zinc (Zn) with the highest nutrient contents being nitrogen (up to 5%), potassium (up to 2%) and magnesium (up to 0.3%) in the harvested dry matter (Sedaghathoor et al., 2009). Mineral nutrients are loosely classified as mobile and nonmobile. Mobile nutrients can move in the plant once deposited and move from older plant part to younger when needed. Nitrogen, potassium, and phosphorus are considered mobile nutrients while calcium, boron, manganese, and iron are nonmobile.

Nitrogen fertilizers are associated with higher yields, and recommendations vary by country but typically range from 100 kg/ha/year to over 600 kg/ha/year (Cheruiyot et al., 2009). Nitrogen deficiency in tea leaves is evident when the leaf content of N drops below 3% which leads to shorter internodes, lighter leaf color, and stunted growth (Hajiboland, 2017). Tea plants take up nitrogen in the form of ammonia (NH_4_^+^) rather than the nitrate form (NO_3_^−^), so nitrate fertilizers are inefficiently absorbed and can even inhibit the entry of ammonia ions and lead to nitrogen fertilizer runoff (Ruan et al., 2007; Yang et al., 2013).

Phosphorus is an essential nutrient in cellular structures and energy metabolism and severe deficiency levels are associated with young and old stem die back. Phosphorus lacks mobility in the soil and its availability is highest in soils with a pH range from 5.5 to 7.0, but limited outside of that range (Hajiboland, 2017). Tea is highly tolerant to phosphorus deficiencies with the most prominent symptom being reduced growth (Nagarajah and Ratnasuriya, 1978). Tea tolerance to low phosphorus may be due to the release of organic acid anions from the roots (Lin et al., 2011). However, the concentrations of flavor- and aroma-related compounds in green tea have been observed to be lower due to phosphorus deficiency (Lin et al., 2012).

Potassium is the last of the major mineral nutrients. Moderate to high soil potassium levels must be maintained for growing tea because, as with nitrogen, harvesting leaves results in the depletion of potassium in the soil (Hajiboland, 2017). Potassium is involved in enzyme activation, carbohydrate metabolism, translocation, and protein synthesis. Sources of potassium fertilizer are potassium chloride (KCl) and potassium sulfate (K_2_SO_4_) but since chloride inhibits nitrogen uptake and reduces theanine levels, which is important for flavor in green teas, the use of KCl is inadvisable (Ruan et al., 2007). Deficiency in potassium is associated with necrosis of the leaf margins primarily at the leaf tips, a depletion of starch in the roots and root die back, which is associated with thin weak branches and a reduction in the size of the leaves (Ruan et al., 2013).

Calcium is a crucial growth regulator and therefore essential to tea plant growth and development (Hepler, 2005). The typical approach to increasing calcium and adjusting soil pH levels is to lime the soil; however, this approach may be difficult in tea gardens due to closely spaced plants. High levels of nitrogen fertilizer can lower the soil pH and one compound used to counter this is to use of the slow-release nitrogen fertilizer, calcium cyanamide (CaCN_2_). In addition to maintaining soil pH balance, CaCN_2_ also limits the growth of soil-borne pathogens and weeds (Oh et al., 2006). The use of calcium cyanamide also reduces the amount of nitrogen lost due to the conversion of nitrogen fertilizer to nitrous oxide (Hirono and Nonaka, 2014). Calcium is not mobile in the plant so deficiency symptoms in tea are expressed as downward curling leaves followed by the appearance of small necrotic spots on the surface of young leaves.

Magnesium is vital for photosynthesis since Mg ions are part of the chlorophyll molecule, and a major enzymatic cofactor for various energy and metabolism processes. Magnesium deficiencies subsequently reduce plant growth and reduce root and shoot translocation. Furthermore, magnesium fertilizer has been shown to increase the uptake of nitrogen ions, increase biomass production, increases amino acid concentrations, particularly theanine in the leaves and roots, and increase of the mobilization of amino acids and sugars in the plant vascular system (Ruan et al., 2012). Magnesium deficiencies are common in acid soils and the symptoms include chlorosis of the leaf margins and interveinal regions alongside green veins, and the turning yellow and red of older leaves.

Sulfur is important in tea production for photosynthesis and is associated with chlorophyll and protein synthesis. Sulfur deficiency can occur in water-logged soils (Dick et al., 2008). These deficiencies are expressed as interveinal yellowing of the leaves, reduced growth of leaves and internodes resulting in shorter and smaller leaves (Ananthacumaraswamy et al., 2003).

Zinc affects the growth and development of tea through gene regulation, enzymatic function, and protein structure. Zinc deficiencies in plants are common and can be induced with excessive phosphorus fertilizers (Mousavi, 2011). Zinc deficiency symptoms in tea plants are expressed as stunting with narrow erect leaves that can form a rosette at the bud apex with reduced chlorophyll contents and photosynthesis (Nelson, 2006). Application of zinc to the soil is highly inefficient, but there has been some successful application of foliar zinc treatments (Huang et al., 2021b). Zinc has also been shown to moderate drought stress by mediating biochemical damage in tea (Upadhyaya et al., 2013).

Tea is an aluminum accumulator species and aluminum content of the leaves is correlated with transpiration rate and duration. About 40–50% of aluminum is partitioned in the cell wall component and boron deficiency increases aluminum binding to cell wall components thus restricting soluble forms from translocating to the phloem and accumulating in the leaves. Maintaining a proper aluminum/boron supply increases the proportion of soluble phenolics in the leaf contributing to the overall quality of the tea (Hajiboland et al., 2015).

Iron and manganese are important micronutrients for maintaining healthy chlorophyll levels in the tea plant which in turn leads to higher quality leaf material and higher yields. The availability of iron is correlated with soil pH, with optimal levels achieved at lower pH. This has led to the use of ferrous sulfate sulfuric acid solutions to lower the soil pH and enhance plant health (Fu et al., 2013). Copper, like iron and manganese, is essential for healthy plant growth but too much can inhibit growth, cause chlorophyll loss and impede photosynthesis. Table 1 shows optimal nutrient compositions in leaves of tea adapted from a Taiwan tea industry extension bulletin (Zee et al., 2003).

**Table 1:** Optimal nutrient compositions in leaves of tea adapted from a Taiwan tea industry extension bulletin (Zee et al., 2003).

Element	Suitable range*	Unit
Nitrogen	4.00–6.00	%
Phosphate	0.25–0.40	%
Potassium	1.50–2.10	%
Calcium	0.25–0.55	%
Magnesium	0.15–0.30	%
Aluminum	400–900	ug/g
Manganese	300–800	ug/g
Iron	90–150	ug/g
Zinc	20–40	ug/g
Copper	8–15	ug/g

### Pests and diseases

*Camellia sinensis* is susceptible to various fungal, bacterial, viral, and algal diseases and to insect, mite, and nematode pests. Many of these specific diseases, the causal pathogens and symptoms are detailed in Supplementary Table 1, which was compiled from multiple sources (Petch, 1923; Hainsworth, 1952; Eden, 1976; Chen and Chen, 1982; Holliday, 1995; Lehmann-Danzinger, 2000; Chakraborty et al., 2006; Bhujel et al., 2016; Wang Y.-C. et al., 2016; Hao et al., 2018; Thangaraj et al., 2018; Koebnik et al., 2021). Fungal plant diseases are the main disease threat to tea with blister blight being one of the most prominent (Lehmann-Danzinger, 2000). All tea plant tissue types are vulnerable to attack by fungal pathogens (Pandey et al., 2021b).

Foliar fungal diseases, or diseases that occur in the leaves, shoots, petioles, and young green stems, are the most economically important because they directly harm the primary product harvested from tea plants. Not only do foliar fungal diseases reduce rates of photosynthesis, severely reducing yield, but also cause significant decreases in flavor volatiles (Ponmurugan et al., 2016). Symptoms of foliar fungal diseases, such as blister blight, caused by *Exobasidium vexans*, or gray blight, caused by *Pestalotiopsis* spp., consist of brown, yellow or necrotic spots or lesions that can spread to encompass the entire leaf (Chen et al., 2017; Pandey et al., 2021b). Defoliation and tip die back are also associated with many foliar diseases in tea, such as anthracnose or brown blight, caused by *Colletotrichum spp*. (Wang Y.-C. et al., 2016). Anthracnose and gray blight have both been reported in Hawaii and California test plots (Zee et al., 2003). Fungal root diseases affect the canopy by reducing water uptake that results in wilt (Gadd, 1936). Wilting of the entire plant as well as yellowing of all the leaves and defoliation are also found in root diseases. Stem cankers and branch/stem diebacks are key symptoms of some root diseases (Sinniah et al., 2017). In some root diseases, the progression of the symptoms, listed above can lead to the death of the stems, branches or the entire plant.

Bacterial pathogens can cause stem canker with exudates, leaf and shoot lesions, and, rarely, stem or crown galls in tea (Uehara et al., 1980; Matsumoto and Fukui, 1998; Tomihama et al., 2009). Viral diseases of tea are associated with yellowing or mottling, chlorotic rings of the leaves, leaf curling, and necrotic black or brown lesions of the phloem (Hao et al., 2018). Algae are not normal plant pathogens but in tea an algal pathogen produces raised spots or blotches along the margins on leaves that can be gray, green, tan, purple to reddish brown in color (Keith et al., 2006).

Insects, mites, and nematodes cause direct damage to tea plants by consuming plant tissue, either through chewing or by feeding on plant sap. Sap-feeding insects and mites can also act as vectors for disease-causing pathogens (Gadd, 1939). Several insect and mite pests have been collected and identified from tea in Hawaii (Hamasaki et al., 2008), including two unique chewing pests and 12 unique sap-feeding pests. While the overall economic losses of tea due to disease is higher than due to insect pests, if left unchecked and given a favorable environment, pests can cause up to 55% yield loss (Lehmann-Danzinger, 2000; Hazarika et al., 2009).

### Pest and disease control

Multiple strategies can be used to control pests and diseases of tea. Standard plant care procedures essential to promoting healthy plants and reducing stress are recommended such as sufficient spacing between plants to permit adequate air flow to reduce humidity and leaf wetness, growing plants in well drained soils, and providing adequate sunlight and nutrients. The reduction of initial inoculum levels of plant pathogens can slow the progression of the disease. This can be accomplished by using chemical controls such as fungicides prior to the growing season, eliminating alternative hosts in or near the fields, or through crop rotation. If chemical controls are used, strict adherence to the manufacturer’s guidelines should be followed. Phytosanitation, or the removal of diseased tissues or plants from the production area, is recommended to eliminate sources of future infections.

Plant activator compounds (PACs) are compounds that stimulate or induce plant defense responses. Yeast extract PACs have been shown to reduce anthracnose and gray mold diseases in Japan, while calcium chloride reduced blister blight disease in India (Yoshida et al., 2010; Chandra et al., 2014). Fungal Biocontrol Agents (FBCAs) are microbial agents that are predicted to have a significant impact on how tea insect pests and pathogens will be managed in the future (Pandey et al., 2021a). FBCAs are a sustainable and eco-friendly method of promoting plant health and stimulating plant growth and defenses (Sain and Pandey, 2017).

Scouting fields for insects and inspecting for crop damage are the primary ways of monitoring pest populations to determine their levels of infestation (Handique and Roy, 2020). Pheromone traps can also be used to monitor for specific pest insects (Noguchi et al., 1981; Mamun and Ahmed, 2011; Srikumar and Radhakrishnan, 2015). Insect, mite and nematode populations can be reduced by using chemical controls, such as insecticides, miticides, or nematicides, prior to the growing season (Barooah, 2011). Eliminating alternative hosts in or near the fields, interplanting non-host plants that inhibit the growth of the pest, and using pheromone traps are key recommendations for control of insect pests of tea (Hazarika et al., 2009).

### Other production problems

Other production problems connected with *C. sinensis* include bud drop, sunscald, and oedema (edema). Bud drop is a developmental condition of flower buds that causes the buds to drop off before opening or that causes the necrosis of young buds. This condition is caused by multiple factors but is mostly associated with plant stresses caused by extreme temperature and moisture fluctuations (Sakai and Hakoda, 1979), nutrient deficiencies (Blake et al., 2021), or by *Camellia* bud mites (Subirats and Self, 1972; Salinero et al., 2008). Sunscald is a leaf disorder that can affect tea plants in full sun and is typically seen as scorched or bronzed areas on the leaves. Sunscald can be a problem on *C. sinensis* plants that are transplanted from shaded areas to locations in full sun (Camellia: Sunburn, 2014; Blake et al., 2021). Oedema is a rupture of leaf cells that occurs on the lower surface of the leaves. This problem occurs when the roots take up excessive water under conditions where foliar transpiration is limited. The excess water in the leaves cause small, light-colored, water-soaked, blister-like areas to form (Camellia: Oedema, 2014). The water-soaked areas or blisters erupt, causing disorganized patches of dead cells that are rusty brown or yellowish brown in color. This condition can occur during the late winter or early spring during cool, cloudy, humid or rainy days (Blake et al., 2021).

## Discussion

With the recent surge in artesian tea production and consumption in the United States there is a renewed interest in the expansion of this specialty crop. In the United States, 17 states now have some form of tea production. In this review, we have tried to pull together the key components of tea production for United States growers. This is not the first-time tea production has expanded beyond its origin in Southeast Asia and, as tea is moved to new environments, production challenges may occur. Management skills, environment modifications and new plant genetic diversity may be required on a regional or national level to meet these challenges and to find economically viable solutions.

Research needs for United States tea production include: a comprehensive analysis of varietal adaptation to environmental stresses, and to different growing regions that can be used to identify the best varieties for each region. There is a need to establish a United States tea germplasm collection with as much genetic diversity as possible to meet the needs of plant breeders and producers for disease and stress resistance and for maintaining and improving quality. A United States specific disease and pest survey should be undertaken to help regional and national efforts to focus on the main production issues. The existing US League of Tea Growers, tea researchers and research institutions working with production issues should link efforts and coordinate national and regional research projects. As United States tea production increases, more resources and attention will need to be focused on managing and improving this specialty crop. Alternative approaches to growing tea in controlled indoor environments including tissue culture and hydroponics should also be expanded as these technologies can alleviate climate related stresses such as drought, fire and flood and enable rapid expansion of lines available for field planting. Moreover, controlled growing environments can be broadly distributed across the US offering high paying technological jobs to a new generation of farmers. Tea is especially well suited for these innovations because presently the United States has no investment in a tea-growing infrastructure. Embracing these new technologies would give tea growers in the United States a unique opportunity to enter the global tea market as high-tech, high yield producers.

## Author contributions

All authors listed have made a substantial, direct, and intellectual contribution to the work and approved it for publication.

## Funding

JG-H acknowledges funding from the National Science Foundation, Chemistry Division, Award: 1758530; UC Davis Interdisciplinary Research Catalyst Faculty Fellows Award and University of California Agricultural and Natural Resources (ANR), 5352:#1704. JL was supported in part by an appointment to the Research Participation Program at the Agricultural Research Service (ARS), USDA, administered by the Oak Ridge Institute for Science and Education through an interagency agreement between the U.S. Department of Energy and ARS.

## Conflict of interest

The authors declare that this review was written in the absence of any commercial or financial relationships that could be construed as a potential conflict of interest.

## Publisher’s note

All claims expressed in this article are solely those of the authors and do not necessarily represent those of their affiliated organizations, or those of the publisher, the editors and the reviewers. Any product that may be evaluated in this article, or claim that may be made by its manufacturer, is not guaranteed or endorsed by the publisher.

## Footnotes

^1^ https://specialtyteaalliance.org/world-of-tea/us-grown-teaand

^2^ https://usteagrowers.com
