# Supplementary material for: United States tea: A synopsis of ongoing tea research and solutions to United States tea production issues
Source: Front Plant Sci. 2022 Sep 23;13:934651. doi: 10.3389/fpls.2022.934651 (PMC9538180; doi:10.3389/fpls.2022.934651)
Supplement: Supplementary file 1 [file Table_1.DOCX]

**Supplementary Table 1. Pathogens, parasites and pests of tea and symptoms they cause.**

| **Bacterial Pathogens** | | |
| --- | --- | --- |
| **Disease** | **Pathogen** | **Symptoms** |
| Bacterial canker | *Xanthomonas campestris* pv. theicola Uehara, Arai & Nonaka  *Xanthomonas gorlencovianum* Daneliya & Tsilosani  *Xanthomonas theicola* | Stem canker or cracking and peeling of the bark results in an open lesion with exudations from the canker. |
| Bacterial shoot blight | *Pseudomonas syringae* pv. thea (Hori) Young Dye & Wilkie | Bacterial leaf lesions formed in association with low temperatures and frost. |
| Crown gall | *Agrobacterium tumefaciens* (Smith & Townsend) Conn | Stem galls at the base of the plant near soil surface. |
| **Viral Pathogens** | | |
| **Disease** | **Pathogen** | **Symptoms** |
| Camellia yellow mottle | Camellia yellow mottle virus | Yellow or creamy white mottled blotches on the leaves. |
| Phloem necrosis | Phloem necrosis virus (Camellia Virus 1) | Necrosis affecting the phloem of the plant with brown discoloration to the leaf vein, leaf curling and twisting, with stunted shoots, necrotic flecking spotting and blotching of the root cambium. |
| Tea plant necrotic ring blotch | Tea plant necrotic ring blotch virus (TPNRBV) | Chlorotic ring blotchy or zonal discoloration of the leaves advancing to necrotic lesions with weak growth. |
| Tea line pattern | Tea plant line pattern virus (TPLPV) | Chlorotic and white zonal discoloration. |
| **Algae Pathogen** | | |
| **Disease** | **Pathogen** | **Symptoms** |
| Red rust (alga) / algal leaf spot | *Cephaleuros virescens* Kunze | Gray, green, tan, purple to reddish brown raised spots or blotches develop along the margins on leaves, a reddish film may also show up on the leaves and stem. |
| **Fungal Pathogens** | | |
| **Disease** | **Pathogen** | **Symptoms** |
| Anthracnose | *Colletotrichum thea-sinensis* (Miyake) Yamamoto *C. aenigma C. endophytica C. truncatum C. camelliae C. cliviae  C. fioriniae  C. fructicola C. karstii C. siamense* | Circular spots appear on the leaves, gradually enlarging, become sunken lesions with round edges, the disease causes wilt, shoot death, and reduced growth. |
| Alternaria blight | *Alternaria alternata* (Fr.) Keissl*.* | Grayish brown patches at the tips and margins of young leaves, that form lesions that extend back toward the midrib causing the leaf to curl, infect leaves with die and defoliate. |
| Armillaria root rot | *Armillaria mellea* (Vahl:Fr.) Kummer *Armillaria heimii* Pegler | Sudden browning of the leaves and the roots may split open. White mycelial mats can be found under the bark at the base of the stem and in the roots. |
| Bird's eye spot | *Cercospora thea* Perth  *Septoria thea* Cavara  *Cercoseptoria thea* (Cavaria) Cruz | Brown to black necrotic spots with a red margin on infected leaves. |
| Black root rot | *Rosellinia arcuata* Petch  *Rosellinia bunodes* (Berk. & Broome) Sacc. | Root and collar rot with black discoloration of the wood, with white mycelia on the roots that turns gray and then black, girdling and canker formation. |
| Black rot | *Ceratobasidium* sp*.*  *Corticium invisum* Petch  *Corticum theae* Bernard | Upper surface of infected leaves turns from reddish brown to yellowish brown to gray. The undersurface of the leaf is covered with a creamy brown mycelia and white patches. Thick fungal cords are produced on the stem and the dead leaves remain attached by the fungal cords. |
| Blister blight | *Exobasidium vexan* Massee | Pale yellow translucent pinhole sized spots that progress to circular blisters mainly on the underside of the leaf that has a dark green water-soaked margin. Blister become velvety white before turn to brown spots, young stems may become distorted and bend or necrotic. |
| Botryodiplodia root rot | *Lasiodiplodia theobromae* (Pat.) Griffon & Maubl. | Dieback of stems and branches. Root and stem-based rot that blackens the vascular tissue. The wood will have a bluish to blackish discoloration. |
| Brown spot | *Calonectria colhounii* Peerally (anamorph: *Cylindrocladium colhounii* Peerally) | Brown spreading spots that expand to the entire leaf. Infected leaves turn black and defoliate. |
| Brown root rot | *Phellinus noxius* (Corner) G.H. Cunningham (anamorph:*Cylindrocladium colhounii* Peerally) | Root rot with white or brown mycelia under the bark, soil particles adhere to the root surface and may be visible on the stem at the soil surface. |
| Brown zonate leaf blight | *Ceuthospora lauri* (Grev.) Grev. | Reddish to greenish brown zonated round lesions surrounded by a dark purple margin form on the periphery of the lower leaves. Black fungal spores are produced in the lesion and the leaves defoliate. |
| Camellia dieback and canker | *Glomerella cingulata* (Stoneman) Spauld. &H. Schrenk (anamorph: *Colletotrichum gloeosporioides* (Penz.) Penz. & Sacc. In Penz.) | Leaves suddenly turning yellow and wilt with branch tip dieback. Grayish irregular lesions form on bark and expand to form sunken cankers. The cankers can girdle the stem resulting in loss of vigor, wilt and death of the plant distal to the canker. |
| Camellia flower or petal blight | *Ciborinia camelliae* | Small, brown, irregular-shaped spots on the flower petals that expand rapidly; whole flower turning brown; flowers dropping from plant, black sclerotia or dried fungal structures form at the flower base. |
| Charcoal stump rot | *Ustulina deusta* (Hoffm.:Fr.) Lind (anamorph: *Ustulina zonate* (Lév.) Sacc.) | Affects plants older than three years of age with a sudden wilt and death of the plant, the leaves remain attached. Black charcoal-like encrustation on the bark at the collar. |
| Collar and branch canker and dieback | *Phomopsis theae* Petch  *Botryodiplodia theobromae*  *Macrophoma theicola*  *Fusarium solani* | Cracks and peeling of the bark, cankers or deep open lesions that are found on the stem, twigs, branches and at the collar region that expand to girdle the stem leading to the death of plant. Affected tea bushes have smaller yellow leaves that prematurely fall. |
| Collar rot | *Rhizoctonia solani* Kühn (teleomorph: *Thanatephorus cucumeris* (Frank) Donk) | Brown to reddish lesions that enlarge to form cankers at the soil surface. Cankers enlarge to girdle the stem causing sporadic wilting that occurs during daylight. |
| Damping-off | *Cylindrocladium floridanum* Sobers & Seymour  (teleomorph: *Calonectria kyotensis* Terashita)  *Hypochnus centrifugus* (Lév) Tul. | Wilted, drying leaves that defoliate with brown to black necrotic lesions on the lower stem and roots. |
| Dieback | *Leptothyrium theae* Petch  *Nectria cinnabarina* (Tode:Fr.) Fr. | Pruning disease, a stem dieback below the pruning point of infected branches, die back branches break off |
| Gray blight / brown blight | *Pestalotiopsis theae* (Sawada) Steyaert  *Pestalotiopsis longiseta* (Spegazzini) Dai et Kobayashi | pale yellow green oval spots are observed on young leaves that progress to irregular round, grayish or necrotic leaf lesions often surrounded by a narrow yellow zone. Black fungal fruiting bodies will form in the center of the lesion followed by defoliation. |
| Gray mold / Botrytis disease | *Botrytis cinerea* Pers.:Fr. | Causes a blight of shoots, buds, young leaves, and flowers with lesions formed with clear margins. Newly emerged leaves are black and shriveled, and infect tissues sporulate with greenish gray conidia. |
| Horse-hair blight | *Marasmius crinisequi* Müller ex Kalchbrenner | Black threadlike structures resembling horse-hair are found on upper branches and are attached to the plant by small brown discs, necrotic leaves drop rapidly from the plant. |
| Leaf necrosis | *Botryosphaeria dothidea* (Moug.: Fr.) Ces. & De Not. | Small brown lesions on young and mature leaves and twigs, that become necrotic with a brown margin. |
| Leaf spot | *Calonectria pyrochroa* (Desmaz.) Sacc.  (anamorph*: Cylindrocladium ilicicola* (Hawley) Boedijn & Reitsma)  *Calonectria theae* C.A. Loos  (anamorph: *Cylindrocladium theae* (Petch) Subramanian *Cochliobolus carbonum* Nelson  *Hendersonia theicola* Cooke  *Pestalotiopsis adusta* Ellis & Everh. *Phaeosphaerella theae* Petch  *Pleospora theae* Speschnew | Small, circular necrotic spots on the leaf margin, central vein, and petiole. Spots enlarge and coalesce, becoming reddish gray in the center and reddish brown at the margins. White fungal mycelia can be observed in the center of the lesion and leaves may wilt. |
| Leaf scab | *Elsinoe theae* Bitancourt & Jenkins | Cork appearance of older infected tissues with scab-like spots, blotches distorted growth. |
| Macrophoma stem canker / twig dieback | *Macrophoma theicola* Petch | Leaves turn brown and droop, symptoms spread to the shoots that become dry and die, the spreading continues to the branch that dies from the tip downward. Swallow, spreading cankers surrounded by thick bark forms on the stem, cankers have black fungal bodies and a white ooze with fungal spores. |
| Net blister blight | *Exobasidium reticulatum* Ito & Sawada | Yellowish green spots form on the upper leaf surface, sometimes with leaf curling, a white-net mycelial mat forms on the underside of leaf opposite the spots. |
| Pale brown root rot | *Pseudophaeolus baudonii* (Pat.) Ryv. | Yellowing of leaves, dieback of branches and wilting of the entire plant, stem collar and roots are covered by a white to bright yellow fungal mycelia. |
| Phoma leaf spot | *Phoma herbarum* | Brown lesions with irregular margins, progress to dark brown lesion edges with a brown or gray center. |
| Phyllosticta leaf spot | *Phyllosticta erratica* Ellis & Everh.  *Phyllosticta theae* Speschnew | Chlorotic to white irregular leaf lesions. |
| Phytophthora root rot | *Phytophthora cinnamon* | Reduced plant growth, chlorosis or yellowing of the leaves followed by the plant wilting and sudden death of the plant, roots may be discolored. |
| Pink disease | *Corticium salmonicolor* Berk. & Broome | Pink colored mat that covers the stem or branch. Causes defoliation and a dieback of the infected branch or stem. |
| Poria branch canker | *Poria hypobrunnea* Petch | Necrotic lesions on the stem  can be associated with pruning. |
| Pythium cutting rot | *Pythium* sp. | Affects root and stem that become watery, whitish brown that progresses to a dark brown color, flatten hollow stems are associated with this disease. |
| Red root rot | *Ganoderma philippii* (Bresad. & P. Henn.) Bresad.  *Poria hypolateritia* (Berk.) Cooke | Foliage of the plant will be a slightly off-green hue with leaves developing a ripened appearance early in the season and leaves on the affect part of the canopy will yellow. On the roots a red mycelial mass that binds to soil particles, |
| Rhizoctonia seedling blight | *Rhizoctonia bataicola* (Taub.) Butl. | Seedling wilt with branch or stem die back. |
| Rim blight | *Cladosporium* sp. | Yellow of the leaf margins progressing to necrosis. |
| Red root rot | *Poria hypolateritia* Berk. ex Cooke | Rapid yellowing of leaves; wilting and sudden death of the plant. The leaves will remain attached to the plant for a limited time. Roots show white or red mycelia and a red discoloration. |
| Root rot | *Cylindrocarpon tenue* Bugnicourt  *Cylindrocladiella camelliae* (Venkataramani & Venkata  Ram) Boesewinkel  *Cylindrocladium clavatum* C. S. Hodges & L.C. May  *Fomes lamaoensis* (Murr.) Sacc. & Trott.  *Ganoderma applanatum* (Pers.) Pat.  *Ganoderma lucidum* (Curtis:Fr.) P. Karst.  *Cylindrocladium parasiticium* Crouss,,M.J. Wingfield, &Alfenas | Diseased cuttings show blighting, drying, and defoliation of leaves with weak axillary bud development. brown to blackish necrotic lesions are found on the stem and cuttings fail to root. |
| Sclerotial blight | *Sclerotium rolfsii* Sacc.  (teleomorph: *Athelia rolfsii* (Curzi) Tu & Kimbrough | Affects the stem at the base of the plant close to the roots; White mycelia will grow from the infected  region producing a dark brown lesion, the mycelia will continue to spread forming a white web on the stem and soil with the formation of black sclerotia. Can cause rapid seedling death. |
| Sooty mold | *Capnodium footii* Berk. & Desmaz.  *Capnodium thea*e Boedijn  *Meliola camelliae* (Cattaneo) Sacc. | Black sooty coating that covers the leaves, can be removed by rubbing, and is associated with aphid and scale feeding. |
| Tarry root rot | *Hypoxylon asarcodes* (Theiss.) Mill. | Sudden die-back of mature bushes, hard black stroma forms at the crown, with thin black lines under the bark. |
| Thorny stem blight | *Tunstallia aculeata* (Petch) Agnihothrudu | Dieback of the branches, fungal fruiting bodies protrude from the dead bark giving a thorny appearance associated with pruning. |
| Thread blight | *Marasmius tenuissimus* (Junghuhn) Singer | Browning of the leaves and twigs. dead leaves hang from the branches by thin fungal threads. |
| Velvet blight | *Septobasidium bogoriense* Pat.  *Septobasidium pilosum* Boedijn & B.A. Steinman  *Septobasidium theae* Boedijn & B.A. Steinman | Violet mycelial growth on the stem and twigs of the plant. |
| Violet root rot | *Sphaerostilbe repens* Berk. & Broome | Found in waterlogged soils affected roots that turn a light purple color with thick irregular purple mycelia under the root bark. Leaves yellow and defoliate. |
| White root rot | *Rigidoporus microporus (*Sw.:Fr.) *Rosellinia necatrix* Berl. Prill. | Disease originates on roots and spreads to collar region, associated with chlorosis or yellowing of leaves, canopy decline, wilt, defoliation and death. |
| White scab | *Elsinoe leucospila* Bitancourt & Jenkins | Scattered irregularly circular spots that are raised above the leave surface that are somewhat smooth with a white or cream center and a tan to brown margin. |
| Wood rot | *Hypoxylon nummularium* Bull.:Fr.  *Hypoxylon serpens* (Pers.:Fr.) J. Kickx  *Hypoxylon vestitum* Petch | Stem cankers with black patches or lines in the wood, infects through wounds, pruning and sunscald injury. |
| Xylaria root rot | *Xylaria* sp. | Roots covered by black mycelial strands that resemble ribbons, necrosis of smaller feeder roots. |
| **Parasitic Nematode** | | |
| **Disease** | **Parasite** | **Symptoms** |
| Burrowing nematode | *Radopholus similis* | Slow decline in growth, chlorosis, premature flowering, and stunted growth. |
| Dagger nematode | *Xiphinema insigne* | Stunted plants, necrotic root lesions, galls at root tips. |
| Lance nematode | *Hoplolaimus columbus* |  |
| Mature tea nematode | *Meloidogyne brevicauda* | Stunted plant growth, wilting, and chlorosis. |
| Pin nematode | *Paratylenchus curvitatus* | Patches of unhealthy plants spindly growth and sparse foliage, leaves are dull and brittle. |
| Reniform nematode | *Rotylenchulus reniformis* | Stunted plants, swollen roots. |
| Root-knot nematode | *Meloidogyne arenaria Meloidogyne hapla Meloidogyne incognita Meloidogyne javanica Meloidogyne thamesi* | Nematodes attack seedlings and young tea roots, brown root lesions, root knot, swollen roots, stunted plant growth, chlorosis, gall formation. |
| Root lesion nematode | *Pratylenchus brachyurus Pratylenchus loosi* | Slow decline in growth, chlorosis, premature flowering, and stunted growth. |
| **Insect and Mite Pests** | | |
| **Common Name** | **Species** | **Symptoms** |
| **Coleophoridae** | | |
| Case-bearer moths / caterpillar | *Coleophora scaleuta*  *Coleophora vigilis* | Leaf caterpillar that consumes the leaves. |
| **Hepialidae** | | |
| Ghost moths / caterpillar | *Endoclita malabaricus Endoclita punctimargo Endoclita purpurescens Endoclita sericeus* | Leaf caterpillar that consumes the leaves. |
| **Geometridae** | | |
| Geometer moths /inch worm / Tea looper caterpillar | *Ectropis crepuscularia*  *Hemithea aestivaria*  *Peribatodes rhomboidaria*  *Biston suppresseria* | Leaf caterpillar that consumes the leaves. |
| Looper caterpillar | *Hyposidra infxaria*  *Hyposidra talaca* | Leaf caterpillar that consumes the leaves. |
| **Noctuidae** | | |
| Turnip moth | *Agrotis segetum* | Leaf caterpillar that consumes the leaves. |
| **Endromidae** | | |
| Bunch caterpillar | *Andraca bipunctata* | Leaf caterpillar that consumes the leaves. |
| **Zygaenidae** | | |
| Red slug caterpillar | *Eterusia aedea* | Leaf caterpillar that consumes the leaves. |
| **Tortricidae** | | |
| Flushworm | *Cydia leucostoma* | Leaf caterpillar that consumes the leaves. |
| **Miridae** | | |
| Tea mosquito bugs  Cocoa mirids | *Helopeltis schoutedeni* Reut.  *Helopeltis antonii* Ghesq.  *Helopeltis theivora* Waterhouse | Adults and nymphs puncture plant cells causing reddish brown lesions, leaves curl and deform, shoots dry up. |
| Scarlet mites and carinate tea mites | *Brevipalpus phoenicis* Geijskes  *Calcarus carinatus* Green | Mites attack tea flushes and leaves, causes a cork-like mass on the underside of the leaf, distorts the growth of young leaves, leaves dry up. |
| Yellow tea mites | *Polyphagotarsonemus latus* | Flush leaves are distorted and cupped with a cork-like brown area between the main veins on the underside of the leaves, flushes turn brown. |
| Red coffee mites  Pink tea rust mites | *Oligonychus coffeae* Nietner  *Acaphylla theae* (Watt) | Discoloration of leaves (yellowish-brown, rusty or purple). Fully developed leaves are attacked. |
| Spider mites (Two-spotted spider mite) | *Tetranychus urticae* | Yellow stippled leaves that may appear bronzed with a webbing cover the leaves. Mites may be visible as tiny moving dots on the webs or underside of leaves, leaves turn yellow and may drop from plant. |
| Chili thrips /yellow tea thrips | *Scirtothrips dorsalis* Hood | Damage caused to buds and young leaves and stems, sandy brown lines form on the underside of the buds and leaves are crinkled distorted and necrotic. |
| Onion thrips | *Thrips tabaci* | Damage caused to buds and young leaves and stems, upward leaf curling. |
| Plague thrips | *Thrips imaginis* | Damage caused to buds and young leaves and stems. |
| Tea green leafhoppers /  Tea jassids | *Empoasca* *(Matsumurasca) onukii* Matsuda  *Empoasca flavescens* | Insects feed on the underside of the leaf causing an uneven downward curling of the leaves, yellow spot. |
| Tea scale | *Fiorinia theae* | Pale yellow spots appear on the leaves which yellow, turn brown and drop prematurely. The scale insect is initially a bright yellow in color but darkens over time to a dark brown and is found on the undersides of leaves. |
| Armoured scale / greedy scale | *Hemiberlesia rapax* | Reduction in stem growth. |
| Tea aphid | *Toxoptera aurantii* | Small soft bodied insects found on the underside of leaves and on stems. The insect’s coloration varies (green, yellow pink, brown, red or black). Leaves may yellow and distort, with necrotic spots and stunted shoots. A sticky, sugary secretion and sooty molds are found on the surface of the plant. |
| Red coffee borer | *Zeuzera coffeae* Nietner | Red to violet brown larva, with yellowish rings bore into the stems and branches causing a dieback. |
| Live wood termites | *Glyptotermes dilatatus* Bug. & Pop.,  *Postelectrotermes militaris* (Des.).  *Microcerotermes* spp. | Insects attack the live stems. causing a dieback. Colonies form in the trunk region. |
| Ground termites | *Macrotermes gilvus* (Hag.) | Insects attack seedling and form colonies in the ground. |
| Tea shoot hole borer | *Xyleborus fornicatus*(Eichoff)  *Xyleborus morigenus* (Blanford) | Insects attack of stems causing a dieback with holes formed in the stem. |
